# Population History Shapes Responses to Different Temperature Regimes in *Drosophila subobscura*

**DOI:** 10.3390/life13061333

**Published:** 2023-06-07

**Authors:** Katarina Erić, Marija Savić Veselinović, Aleksandra Patenković, Slobodan Davidović, Pavle Erić, Marina Stamenković-Radak, Marija Tanasković

**Affiliations:** 1 Department of Genetics of Populations and Ecogenotoxicology, Institute for Biological Research “Siniša Stanković”—National Institute of the Republic of Serbia, University of Belgrade, Despot Stefan Blvd. 142, 11060 Belgrade, Serbia; aleksandra@ibiss.bg.ac.rs (A.P.); slobodan.davidovic@ibiss.bg.ac.rs (S.D.); pavle.eric@ibiss.bg.ac.rs (P.E.); marija.tanaskovic@ibiss.bg.ac.rs (M.T.); 2 Faculty of Biology, University of Belgrade, Studentski Trg 3, 11000 Belgrade, Serbia; marijas@bio.bg.ac.rs (M.S.V.);

**Keywords:** *Drosophila subobscura*, inversion polymorphism, Hsp, thermal stress

## Abstract

*Drosophila subobscura* is considered a good model species for investigation of a population’s ability to adapt and cope with climate changes. Decade long research has shown that inversion frequencies change in response to environmental factors indicating their role in adaptation to novel environments. The mechanisms behind organisms’ responses to temperature are complex, involving changes in physiology, behavior, gene expression and regulation. On the other hand, a population’s ability to respond to suboptimal conditions depends on standing genetic variation and population history. In order to elucidate the role of local adaptation in population response to the changing temperature, we investigated the response to temperature in *D. subobscura* individuals originating from two different altitudes by combining traditional cytogenetic techniques with assessing the levels of Hsp70 protein expression. Inversion polymorphism was assessed in the flies sampled from natural populations and in flies reared in laboratory conditions at three different temperatures after five and sixteen generations and Hsp70 protein expression profile in 12th generation flies at the basal level and after heat shock induction. Our results indicate that local adaptation and population history influence population response to the changing temperature.

## 1. Introduction

Chromosomal inversions are traditionally described as adaptive structural mutations that become advantageous in populations by capturing favorable allele combinations [1]. Since Dobzhansky’s pioneering work indicated the adaptive significance and role of environmental cues in shaping inversion variability [2,3,4], the experimental evidence has grown, but mechanisms of generating and maintaining inversion frequencies in populations are still a matter of scientific debate. There are several proposed concepts for explaining their presence and perseverance, including the Dobzhansky coadaptation hypothesis [5], Wasserman supergenes concept [6], the Kirkpatrick and Barton [7] local adaptation scenario, the positive selection hypothesis [8], and concepts that define inversion as a locked region of a chromosome which may be adaptive or neutral [9]. Each of these concepts is described and documented, many of them in *Drosophila* species, most of them having in common that due to the absence of recombination in heterokaryotypes, each inversion captures specific alleles in a beneficial combination, and as such, their frequency changes in response to the environment.

Regardless of mechanism, environmental variables found to influence changes in inversion polymorphism are many. Climatic factors such as temperature, humidity, precipitation and isolation are the most common factors correlated with shifts in inversion distribution in natural populations [10,11,12], but heavy metal pollution [13] and environmental heterogeneity are also identified as selective pressures shaping this type of genetic polymorphism. The adaptive value of rich chromosomal inversion polymorphism in *Drosophila subobscura* is particularly well documented, with numerous evidences of long-, mid- and short-term changes in their frequencies correlating with environmental variables. Parallel latitudinal distribution in inversion clines [14] and increased frequencies of previously considered heat-adapted inversions in the northern area of species distribution [10,15,16], identified temperature as a primary environmental factor driving these changes simultaneously demonstrating the species’ ability for rapid adaptation to global warming [16,17,18]. Despite strong evidence that inversion polymorphism in natural populations is strongly associated with climatic factors, the causal nature of this association in laboratory conditions has not been confirmed, and the results of these studies are largely inconclusive [19,20], with the main explanation being that stable laboratory conditions could not reflect the complexity of the natural environment. Recent research indicates that the synergistic effects of factors related to temperature and precipitation, as well as environmental heterogeneity, demographic factors, and population history, have a considerable contribution in shaping the arrangement frequency patterns in *D. subobscura* natural populations [11]. 

By origin, *Drosophila subobscura* is a Palearctic, temperate region species, with a thermal range between 6 and 26 °C, but it is generally considered a cold-adapted species with a thermal optimum of 18 °C [21]. With more than 60 described inversions and about 90 different chromosomal gene arrangements across five acrocentric chromosomes, it is one of the species with the richest inversion polymorphism in the *Drosophila* genus [22,23,24]. Standard chromosome arrangements are generally associated with adaptation to colder climates, while complex arrangements are more frequent in warmer areas of distribution [15,18,25]. The O chromosome, with about 40 described inversion arrangements [21], is considered as a prime candidate for thermal adaptation but some studies have shown that chromosome E also has a large effect on heat resistance [25]. Various experiments confirmed the O chromosome as the location of several members of the Hsp70 protein family [9,26,27] as well as several candidate genes associated with thermotolerance [28], but all chromosomes have differentially expressed genes associated with temperature response [27]. Heat shock proteins are one of the highly conserved chaperone family that have a big role in reducing cellular damage during thermal stress [29,30]. The heat shock proteins Hsp70s induced by high temperature are considered essential for surviving high-temperature-induced stress [31]. Sequencing has confirmed that in *D. subobscura* the Hsp70 family consists of two intronless and closely spaced Hsp70 genes arranged as a head-to-head inverted repeat (Hsp70IR), and that this gene family has identical genomic organization in Ost and O_3+4+7_ karyotypes [9,32]. Experiments conducted on homokaryotic *D. subobscura* strains revealed that O_3+4_ flies have higher basal expression levels of Hsp70 proteins not boosted after heat shock and that Ost individuals show a different pattern [33]; however, these results could not be confirmed in another experiment comparing basal Hsp70 levels in homokaryotic O_3+4_, O_3+4+7_ and Ost files from different geographical regions [32]. Although specific genomic organization and copy number variation of the Hsp70 gene family still remains elusive and most probably population-specific, nucleotide variation at the Hsp70IR locus showed higher identity between Hsp70 gene paralogs of the same inversion than between orthologues of different inversions [9]. However, even small changes in temperature can induce heat shock response mediated through Hsp70 and several studies have shown that in some *Drosophila* species adaptation to colder climates strongly influences the magnitude of that response, indicating that changes in regulatory regions may be a target of selective pressure. Other experiments have demonstrated genome-wide changes in inversion polymorphism in response to temperature [16] and differentially expressed genes dispersed across all chromosomes [27], highlighting the complex mechanism behind organisms’ response to temperature changes. Advancements in long read sequence technology allow for finding and locating structural and functional genes. This also provided a means to isolate and characterize breakpoint sequences necessary to elucidate the role of inversions in adaptation to climate change. The analysis of breakpoint sequences for 12 inversions in *D. subobscura* associated with thermotolerance dispersed across all chromosomes has revealed that breakpoints do not disrupt any candidate gene directly associated with temperature adaptations, thus further corroborating the genetic complexity of thermal traits [34,35,36,37,38,39,40,41]. 

Although molecular genetic analysis has provided deeper insights into genes and mechanisms involved in temperature adaptation in this species, many evolutionary issues still remain unresolved. Despite the power of sequencing technology for the characterization of inversion polymorphisms, the cytogenetic technique, although with lower resolution, still remains a valuable tool for the analysis of the adaptive significance of this structural chromosomal polymorphism.

Our previous work on the effect of population history and different laboratory thermal conditions, performed with populations from different altitudes, indicated that both factors shape responses to stressful conditions [42]. In the [42] study, we analyzed four stress resistance traits (desiccation, starvation, chill coma recovery time, and heat knock-down resistance) and found indications of population-specific responses. Individuals from higher altitude tend to cope better in the extreme experimental environments since they have better heat knock-down resistance and chilly coma recovery time, while individuals from lower altitude performed better in desiccation and starvation assays. In the present paper, we used the same laboratory populations, i.e., 42, with the aim of investigating the mid- and long-term responses to temperature in *D. subobscura* individuals originating from two different altitudes by combining traditional cytogenetic techniques with assessing the levels of Hsp70 protein expression. Inversion polymorphisms were assessed in the flies sampled from natural populations and after 5 and 16 generations in laboratory conditions at three different temperatures. The Hsp70 protein expression profile was performed on the 12th generation at the basal level and after heat shock induction. Laboratory temperature regimes were chosen as stressful but not lethal for *D. subobscura*, with 16 °C prolonging development time, 19 °C considered as optimal, and 25 °C as a temperature which may induce male sterility [21,25]. The main question was to reveal if local adaptation shapes population responses to changing temperatures as one of the climate parameters.

## 2. Materials and Methods

### 2.1. Fly Samples

Wild caught females were sampled in mid-August on Stara planina mountain, in Serbia, from two different altitudes: 1080 m and 1580 m (43.395255° N; 22.603995° E and 43.374145° N; 22.618110° E, respectively). After establishing isofemale lines (IF) in the laboratory, their progeny (about three to five females and males per each line) was mixed to make two mass laboratory populations that differ in sampling location: high altitude (marked as H) and lower altitude (marked as L). The first generation progeny of these two laboratory mass populations was used to establish three experimental groups per population which differed in their maintenance temperature: 16 °C, 19 °C and 25 °C. There were six experimental groups in total, reared on standard Drosophila medium (water/cornmeal/yeast/sugar/agar/nipagine), at approx. 60% relative humidity, light of 300 lux and 12/12 h light/dark cycles.

### 2.2. Chromosome Preparations and Analysis

Inversion polymorphism analysis was performed with wild *D. subobscura* males or F1 males of individual females from natural populations and with those from F5 and F16 generations, in all experimental groups from different temperature regimes. Each unique cross between 4 and 5 virgin females from the Küsnacht laboratory strain and between 25 and 35 males from each sample was performed. The Küsnacht strain was used to identify chromosomes with inversions in the progeny since it is homokaryotypic for the standard chromosomal configurations on all five chromosomes (A_ST_, J_ST_, U_ST_, E_ST_, and O_ST_). The sample size of the studied populations is given in Table 1. For chromosome preparation, third-instar larval salivary glands were crushed, and chromosomes were stained with an aceto-orcein solution. The Kunze–Mühl and Müller chromosome map [43] were utilized for the cytological examination of chromosome configurations. The chromosomes of 10 third-instar larvae from each cross progeny were examined in order to increase the accuracy in defining the karyotype of the crossing male.

### 2.3. Heat-Shock Assay

Experimental groups established twelve generations earlier were maintained at the above-mentioned temperature regimes and conditions. Their female and male freshly emerged progeny was collected and separated in order to prevent mating. For each assay, approximately 50 ♀ and 50 ♂ virgin flies were used per experimental group. Five flies were placed into empty falcon tubes (50 mL volume) with moistened plugs to prevent desiccation and placed in an incubator; set to 37 °C for 90 min. Flies were allowed to recover at room temperature for 30 min and then frozen at −80 °C for further analysis. To measure basal levels of Hsp70 expression, the same number of flies from each experimental group were collected and frozen in the same way.

### 2.4. Hsp70 Expression Quantification by ELISA (Enzyme-Linked Immunosorbent Assay)

A total of six biological replicates per group were analyzed. All protocols were performed according to the manufacturer’s instructions. Homogenizations of five flies per experimental group and per sex (in total, 12 for control and 12 for heat shock) were performed in 1.0 mL of a suitable mix (Sigmafast Protease inhibitor tablet, Sigma-Aldrich Co., St Louis, MO, USA) following the protocol in [44]. The total protein concentration was determined by BCA assay (Sigma-Aldrich Co.) in 96-well microplates with 10 μL quadruplicate samples. Standardization was performed against diluted bovine serum albumin. For the ELISA assay (Sørensen et al. 1999) a protein concentration of 50 mg/mL per sample was used. Hsp70 concentrations were quantified using a Hsp70-specific monoclonal primary antibody (clone 5A5, dilution 1:1000 PBS; Thermo Scientific Inc., Bremen, Germany) and a HRP-conjugated secondary antibody (dilution 1:2000, anti-mouse IgG; Thermo Scientific). A spectrophotometer microplate reader (Multiscan Specrtum, Thermo, Germany) was used to measure color reactions at 490 nm, and each plate was organized as follows: 24 randomly distributed samples (12 heat-shock and 12 control) with three replicates and one blank (without primary antibody) per sample. A blank is commonly used to allow corrections for a non-specific signal. In order to obtain the correction factor for all plates, we used the standard value of one randomly chosen plate established as a reference. 

### 2.5. Statistical Analysis

Population genetic analysis was based on the frequencies and distribution of different inversions. For the analysis of differences in individual chromosome arrangements we used Z statistics. Observed (H_O_) and expected (H_E_) heterozygosity, were calculated using Arlequin ver. 3.5.2.2 software under the Hardy–Weinberg equilibrium model [45]. The same software was used to estimate the parameters of genetic diversity (number of gene copies, number of alleles/inversions), pairwise populations *F_ST_* values and Nei’s distances. The statistical significance of all performed tests was assessed with 10,000 permutations. The matrix of pairwise population *F_ST_* values was visualized using a multi-dimensional scaling method (nonmetric MDS) implemented in the PAST 4.03 software [46] and the R functions connected with Arlequin software. The Hardy–Weinberg equilibrium was tested using Arlequin with 1,000,000 steps in MC and 100,000 dememorization steps. 

For the discriminant analysis of the principal components (DAPC) we used karyotypes of each individual. This method consists of performing the linear discriminant analysis (LDA) on the principal components analysis (PCA)-transformed matrix. LDA was performed on the first 14 PCs, cumulatively conserving 98.9% of the total variance. Additionally, the PCA-transformed matrix was used to find the optimal number of clusters using Ward’s method. We tested 2–50 clusters, and the optimal number of clusters was chosen using BIC statistics using the “diffNgroup” method. 

The relative expression of Hsp proteins was analyzed using the full factorial general linear model (GLM) procedure and Fisher’s post hoc test in the STATISTICA ver. 12 with fixed factors: Population, Rearing temperature, Treatment and Sex.

## 3. Results

### 3.1. Inversion Polymorphism

Standard parameters of genetic diversity per group are shown in Table S1 and pairwise comparisons with Z values in Table S2. The highest observed heterozygosity (H_O_) was detected in the H natural population, with a general trend of higher H_O_ in natural compared to laboratory populations, and a weaker trend of higher H_O_ in laboratory populations derived from H then its L counterparts. Inversion polymorphism frequencies are shown in Table S3. Based on the Z statistic, inversion frequencies in natural populations differ, especially for A, J and O chromosomes (Table S2). In both natural populations, O_3+4_ is the most frequent arrangement for the O chromosome, but with significantly lower frequency in the L population. Complex arrangements in the O and E chromosomes are mostly absent from the L lab populations but retain small frequencies in the H lab populations regardless of temperature conditions. It is interesting to note that arrangement frequencies in the J chromosome behave differently in the H and L populations. While Jst in the H natural population has a much lower frequency than J_1_, its frequency rises in laboratory conditions, but the opposite trend is observed for the L population where J_1_ is dominant in the natural population and preserves its frequency in laboratory conditions. In the H population, after five generations at different temperatures, the Ost is more frequent in warm conditions, but after 16 generations of standard arrangements in A, J and O chromosomes have significantly higher frequencies in both cold and optimal conditions compared to the warm. In the L population after five generations, there are no differences in Ost frequencies at different temperatures, but it is more frequent in warm conditions after 16 generations.

**Figure 1:**
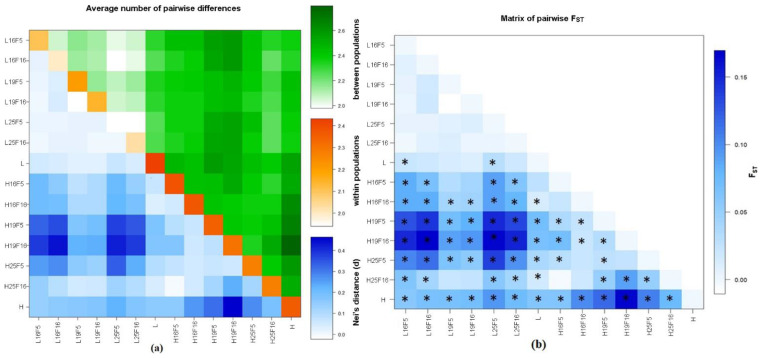
Matrixes of the average number of pairwise Nei’s (**a**) and *F_ST_* (**b**) distances based on the analysis of chromosome arrangement distributions in two populations from different altitudes in three different temperature regimes across generations; (**a**) the average number of pairwise differences between populations is presented above diagonal, the average number of pairwise differences within the population is presented diagonally, and Nei’s distances are presented below diagonal. (**b**) Statistically significant *F_ST_* values are marked with an asterisk (*).

Nei’s distances (Figure 1a) and *F_ST_* statistics (Table S4, Figure 1b) show that differentiation among experimental groups in the H population is higher than in the L population. While it appears that different temperature regimes do not influence chromosome arrangement distribution in the L population, the H population reveals that flies reared in optimal conditions exhibit different arrangement combinations than flies reared at higher and lower temperatures. The highest *F_ST_* values were noted between the H and L comparisons, suggesting different mechanisms of response to suboptimal temperature conditions (Table S4).

**Figure 2:**
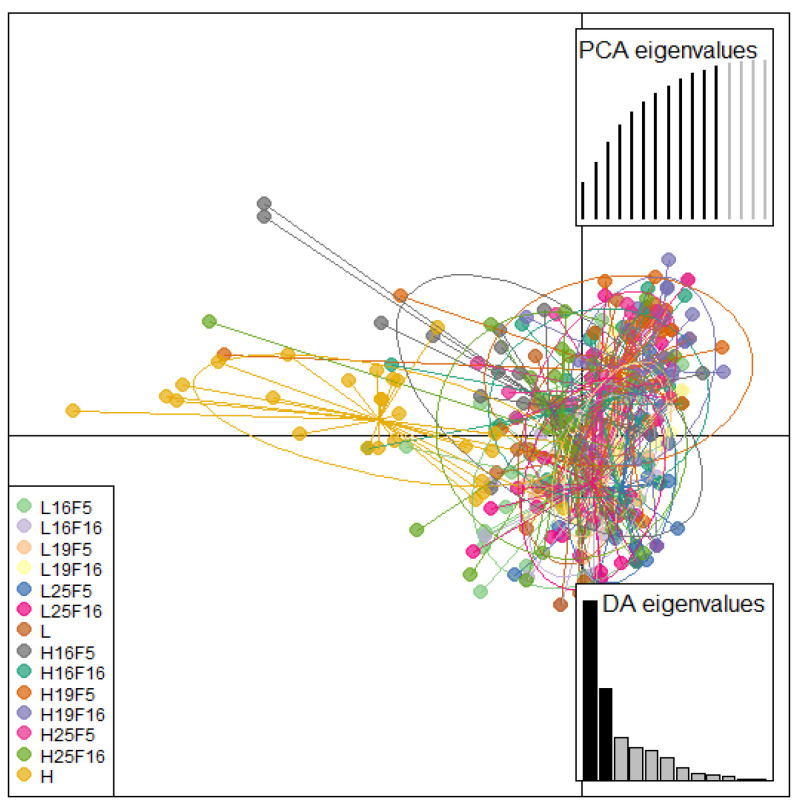
Discriminant analysis of principal components. The first and second linear discriminants are presented in the plot.

**Figure 3:**
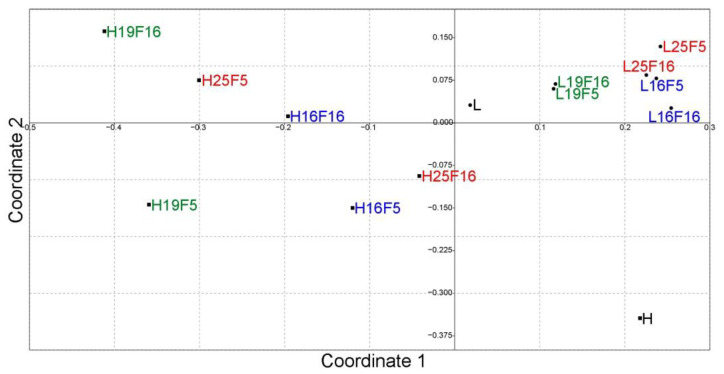
Non-metric multi-dimensional scaling plot of *F_ST_* distances between populations. The goodness of fit is expressed with the stress value which is 0.09865 for this dataset.

Discriminant analysis of principal components (DAPC) with an inferred number of clusters and multi-dimensional scaling (MDS) showed a similar pattern of population differentiation (Figure 2, Figure 3 and Figure S1). The natural population from the higher altitude occupies the most distant position from all other analyzed groups and while there is a considerable variability within groups some patterns can be observed. Laboratory populations originating from the lower altitude tend to cluster together depending on the laboratory’s thermal conditions and temperature, most notably for optimal laboratory temperature. Laboratory populations originating from the higher altitude are more dispersed across the PCA landscape with observable differentiation according to laboratory conditions and generations.

When the H and L populations were analyzed separately, strong differentiation could be observed between the natural populations and corresponding groups reared in laboratory conditions, and such differentiation was more prominent in populations from the higher altitude (Figures S2–S5, Table S5a,b).

### 3.2. Hsp70 Expression

**Figure 4:**
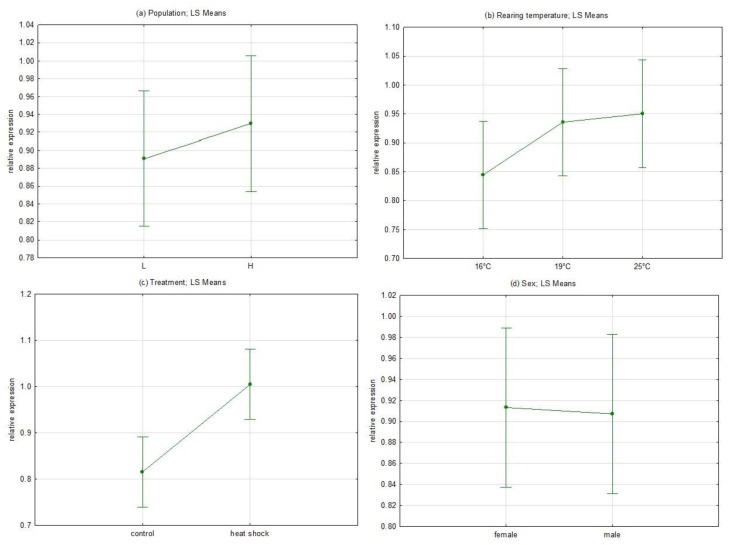
Full factorial GLM analysis with fixed factors (**a**) Population, (**b**) Rearing temperature, (**c**) Treatment and (**d**) Sex.

The results of the relative Hsp expression in each experimental group are presented in Table S6. The highest expression was observed in heat shock-treated females reared at 19 °C from the H population. The results of full factorial GLM analysis with fixed factors as Population, Rearing temperature, Treatment and Sex are shown in Table S7 and Figure 4. Among the analyzed factors, only Treatment showed a statistically significant influence on the level of Hsp expression (F = 12.205, *p* = 0.000668). Besides this, individuals in the H group tended to have higher levels of expression, rising with temperature with no difference between sexes.

**Figure 5:**
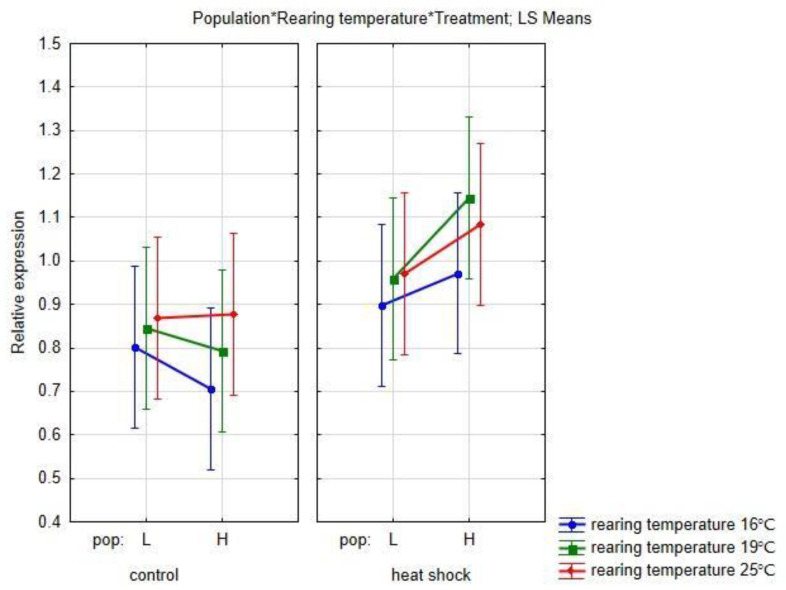
Results of the full factorial GLM analysis with fixed factors population, rearing temperature, and treatment.

Although no significant interaction was observed between the combinations of factors, it is interesting to note the results of Fisher’s post hoc analysis of population/rearing temperature/treatment (Figure 5 and Table S8). Individuals from the H population reared in colder conditions (16 and 19 °C) had significantly higher levels of Hsp expression when exposed to the extreme temperature than flies reared at the same temperature without heat stress. The same is not observed in individuals originating from the L population.

## 4. Discussion

Abundant inversion polymorphism *D. subobscura* has been in the focus of scientific research for over seven decades, as one of the first genetic variability markers linked to a species adaptation to climate change [47]. Although most studies associate changes in inversion frequencies across latitude, seasons and over long time periods directly with temperature [16,48], recent studies have indicated that temperature could not be the only factor driving these changes [11]. Natural environments are complexes of interrelated, fluctuating factors with frequent changes in both intensity and direction. Inversion polymorphism in *D. subobscura* is considered to be adaptive [10,13,18,47]. However, mechanisms of generating and maintaining inversion frequencies in populations as well as a genetic basis for their adaptive potential still remain elusive, making them scientific old ladies with quite a few secrets and never out of trend.

We attempted to experimentally validate the decade-old assumption that temperature is the main force shaping the distribution of chromosomal inversion frequencies across latitudes and climates by maintaining two distinct populations of *D. subobscura* from two altitudes in constant laboratory conditions under three thermal regimes, and assessing inversion polymorphism change over generations. Furthermore, we assessed whether adaptation to different thermal regimes in laboratory conditions influences the expression of the Hsp70 protein, both on a basal level and after heat shock.

Under the assumption that temperature was the main selective factor forming latitudinal clines [16,25,47], chromosome frequencies in laboratory populations should mimic those clines, i.e., populations reared in warm laboratory conditions should resemble natural populations in warmer parts of species distribution. Standard chromosome arrangements are more frequent in cold conditions, and the frequency of complex chromosomal arrangements rises with temperature [10,15,18,49,50]. Laboratory groups derived from the H population after 16 generations exhibited a similar pattern of chromosomal arrangement frequencies as natural populations, with Ost frequencies decreasing and O_3+4_ increasing in warm conditions. In laboratory groups derived from the L population, no such pattern could be detected, and Ost frequency was elevated in warm conditions. For other chromosome arrangements associated with thermal response [51], no clear pattern in frequency changes could be observed with some cold-adapted populations being more frequent in warm conditions and vice versa and with a population-specific footprint. Our results of inversion frequencies in different temperature regimes did not match with the results obtained from previous studies in natural populations, but are in concordance with the results of several previous studies in *D. subobscura* [19,20], suggesting that either laboratory settings cannot fully replicate changeable natural conditions or temperature alone could not be accountable as the main evolutionary force shaping inversion clines although its significant influence could not be neglected [25]. Previous attempts to replicate natural conditions in the laboratory provide various explanations, from laboratory simplicity versus nature complexity, extreme and unpredictable fluctuations of environmental factors to rather indirect than direct effects, and warrant extreme cautions in extrapolating laboratory data to nature [19,25,52,53]. Our results, based on the differences in population response, suggest that response to temperature changes may be population-specific, and that some inversions may be highly locally adapted containing different sets of adaptive alleles [7,54,55,56,57]. Moreover, our results indicate that the observed frequencies in laboratory conditions after different temperature regimes depend on population history, population initial conditions, and local environment [25,58,59,60]. 

Further evidence that population history may have an effect on how the population responds to temperature changes is observed in differences in population structure. Our chosen natural populations from different altitudes across the mountain slope of Stara planina showed initial differences in inversion polymorphism, and that differences are preserved across generations and laboratory conditions with high *F_ST_* values. A similar pattern was observed at the chromosomal level by [20], where two distinct populations with different geographical origins remained differentiated after 40 generations in laboratory conditions, as well as at the genome-wide level at SNPs tested for signs of selection [61]. In addition to the observed differentiation, our chosen populations behaved differently in laboratory settings. While there is no observable differentiation among the L experimental groups across generations and temperature regimes, in the H experimental groups there was differentiation between groups reared in optimal and both cold and warm conditions, but interestingly not between groups reared in cold and warm conditions. This could indicate that extreme climate conditions in higher altitudes enhance variability and enable a more plastic response when populations are faced with suboptimal temperatures. Not only that, the H population was the most distant from all other experimental groups with the highest mean heterozygosity, but all groups derived from H had a higher heterozygosity than their L counterparts. The rare arrangements on the O and E chromosomes were more frequent in the H experimental group and there was a striking difference in the J chromosome arrangement frequency change, with Jst having higher frequency in H groups and J_1_ in L groups, irrespective of the thermal regime. Higher heterozygosity may be associated with the distribution of differentially expressed genes dispersed across all chromosomes [27], providing different mechanisms for coping with temperature challenges.

The observed changes in the Hsp protein expression patterns are in line with the observed differences between the H and L populations in laboratory conditions based on inversion polymorphism. Although not significant, the observed increase in Hsp70 levels with rising temperature as well as different patterns of expression in the H and L population further confirm the influence of population history. Expression levels are lower in H groups without temperature stress, but after heat exposure, all H groups have higher expression levels compared to their L counterparts, indicating a better ability to alleviate the influence of the extreme rise in temperature. Moreover, thermal shock induces higher protein expression, but significant difference between the basal- and temperature-induced levels could be observed only in H groups reared in cold and optimal conditions. Hsp protein expression is a balance between their protective role in various stressful conditions [62,63] and their high-energy demands, which may lead to reduced fitness under moderate and non-stressful conditions [30,64,65,66,67]. Although some results show that warm-adapted individuals tend to have a higher temperature threshold with increased Hsp70 protein and Hsp70 mRNA levels after heat stress compared to cold-adapted ones [68,69,70,71,72], several studies have reported the opposite trend [62,73]. In our experiment, all individuals reared in warm conditions tended to have higher Hsp70 expression than individuals reared in cold or optimal conditions, but Hsp70 expression after heat shock appeared to be population-specific, with a more pronounced rise in population from fluctuating climate factors in natural settings. The indication that H individuals have a greater capability to cope with extreme temperature stress has been previously shown in [39], where H individuals exhibited significantly longer heat knock-down time than L individuals. However, we cannot exclude plasticity in thermal response as a factor contributing to observed differences between H and L populations. Although plasticity of thermal response in *Drosophila* species is not ubiquitous [74,75,76,77,78], in *D. subobscura* there is clear evidence of a plastic response to thermal variation [79].

Recent studies with *D. subobscura* showed the presence of population variation in evolutionary potential for long-term thermal adaptation suggesting that environments with higher thermal variation and pronounced thermal extremes may enhance reproductive success at higher temperatures [80]. Our results indicate that population history and extreme environmental conditions may have more impact on shaping a population’s ability to cope with changing environments, whether they are optimal or suboptimal. A natural population from a harsher environment (H) is more genetically distant from both a more stable natural population (L) and different laboratory populations, and selection in laboratory shapes response of that population towards better coping with different thermal stressors. Results of Hsp expression indicate similar relations. While in the L population there is no significant difference between levels of Hsp protein between control conditions and the heat shock assay, in the H population the level of Hsp significantly rises under extreme temperature stress.

Climate change and rising temperatures strongly influence changes in the genetic structure of natural *D. subobscura* populations, pushing them to their upper thermal limits, but the contribution of some other factors, such as population history, has to be considered. The results obtained in our study reveal that local adaptations significantly shape the populations’ response to the changing temperature as one of the climate parameters. Combined with phenotypic assays and functional expression of relevant proteins, chromosomal inversions and frequency changes of gene arrangement seem to be a relevant marker for inferring population structure, differentiation, and ability to withstand environmental challenges, such as temperature stress.

## Figures and Tables

**Table 1:** The sample size of the studied populations.

Population H	*n*	Population L	*n*
Wild-caught males	29	Wild-caught males	25
F5 at 16 °C	31	F5 at 16 °C	31
F16 at 16 °C	31	F16 at 16 °C	33
F5 at 19 °C	34	F5 at 19 °C	30
F16 at 19 °C	28	F16 at 19 °C	29
F5 at 25 °C	29	F5 at 25 °C	28
F16 at 25 °C	30	F16 at 25 °C	32

## Data Availability

The data presented in this study are available in article.

## Supplementary Materials

The following supporting information can be downloaded at: https://www.mdpi.com/article/10.3390/life13061333/s1, Figure S1: Number of inferred clusters; Figure S2: *F_ST_* distances based on the analysis of chromosome arrangements between groups derived from lower altitude, L; Figure S3: *F_ST_* distances based on the analysis of chromosome arrangements between groups derived from higher altitude, H; Figure S4: Discriminant analysis of principal components for the population originating from higher altitudes; Figure S5: Discriminant analysis of principal components for the population originating from higher altitudes. Table S1: Values for the analyzed parameters of genetic diversity; Table S2: Pairwise comparisons with Z values; Table S3: Frequencies of chromosomal inversions in L and H populations, reared at three different temperatures, across generations; Table S4: *F_ST_* statistics for L and H populations, *p* values are presented above diagonally; Table S5: Pairwise population *F_ST_* for L and H groups; Table S6: Relative Hsp expression in each experimental group; Table S7: Full factorial GLM analysis of Hsp expression with fixed factors: Population, Rearing temperature, Treatment and Sex; Table S8: Fisher’s post hoc analysis of population/rearing temperature/treatment.
