# Supplementary material for: Population History Shapes Responses to Different Temperature Regimes in Drosophila subobscura
Source: Life (Basel). 2023 Jun 7;13(6):1333. doi: 10.3390/life13061333 (PMC10300762; doi:10.3390/life13061333)

Figure S1. Number of inffered clusters.

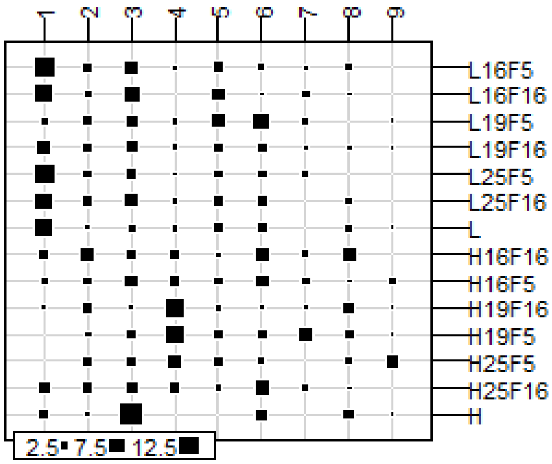

Figure S2. F_{ST} distances based on the analysis of chromosome arrangements between groups derived from lower altitude - L.

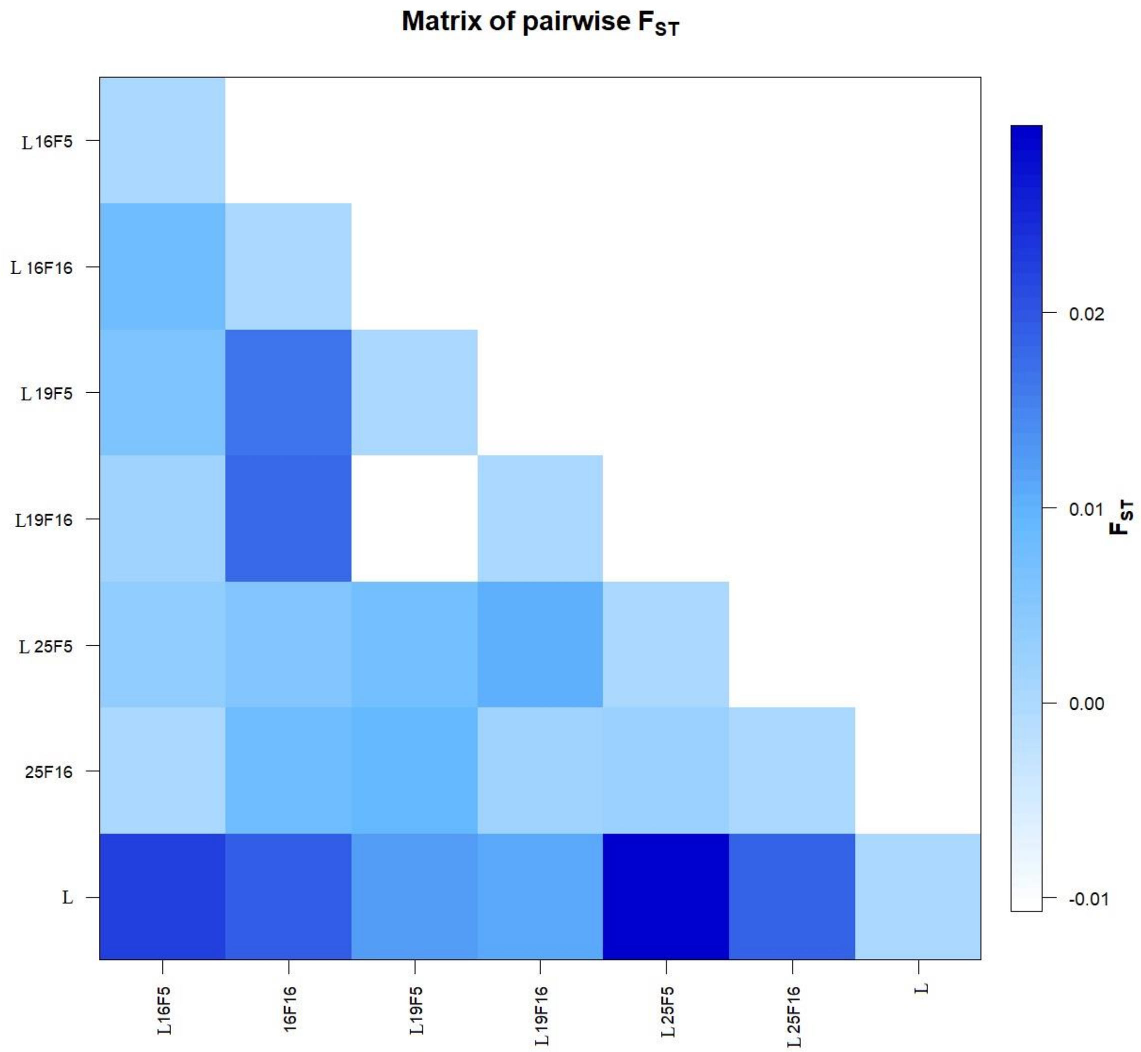

Figure S3. *F_{ST}* distances based on the analysis of chromosome arrangements between groups derived from higher altitude - H.

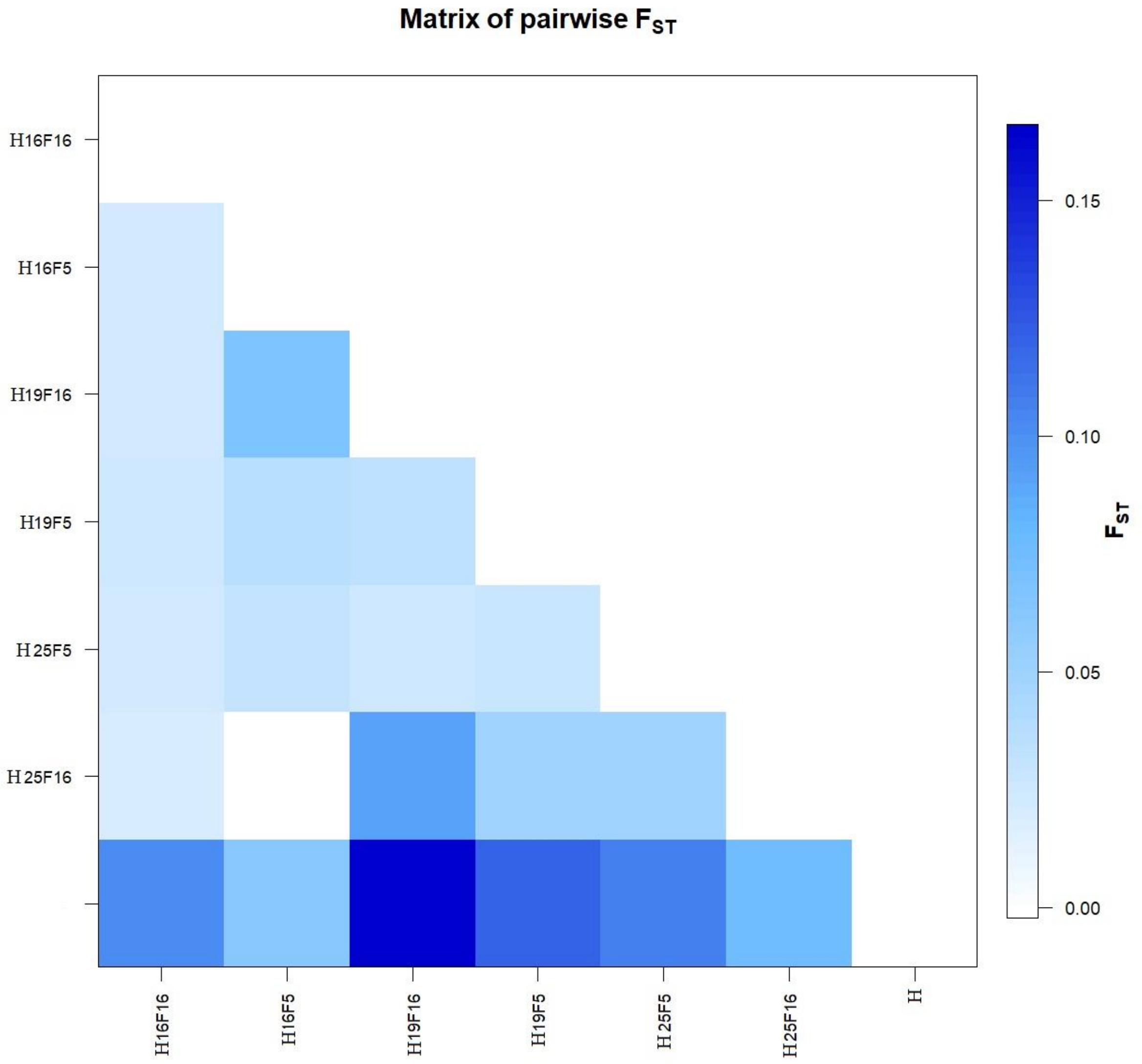

Figure S4. Discriminant analysis of principal components for the population originated from higher altitude.

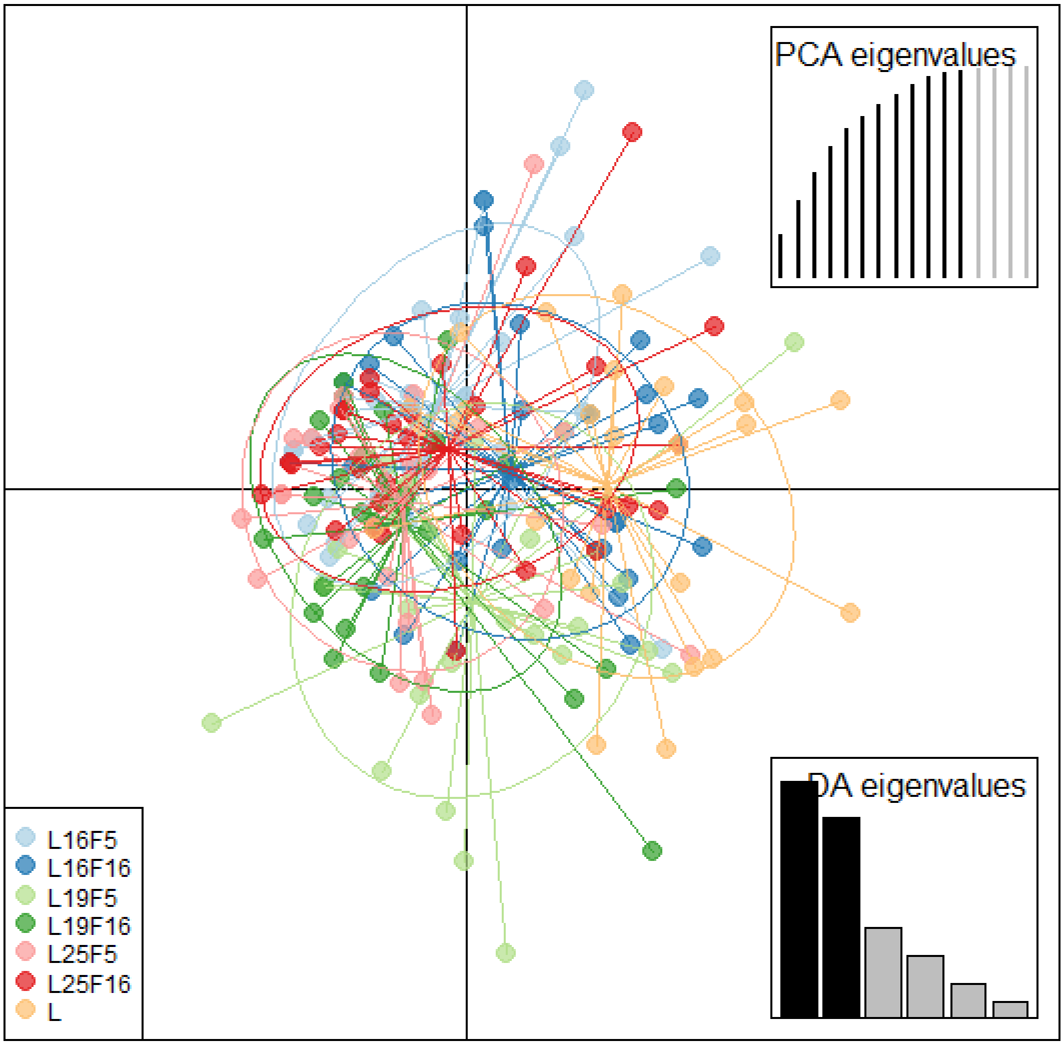

Figure S5. Discriminant analysis of principal components for the population originated from higher altitude.

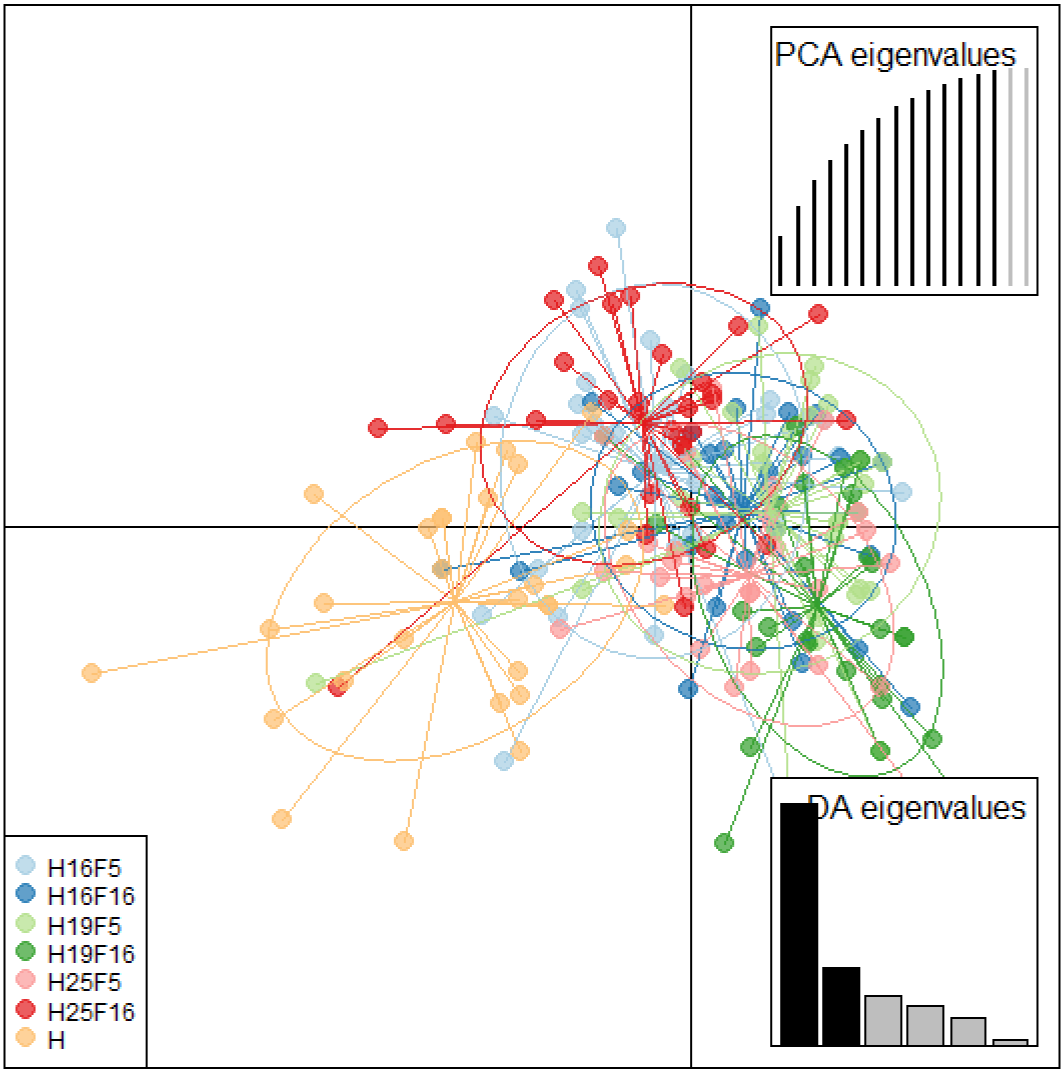

Supplement: Supplementary file 1 [file life-13-01333-s001.zip › Suplementary figures.pdf]
