# Supplementary figures and images for: Population History Shapes Responses to Different Temperature Regimes in Drosophila subobscura
Source: Life (Basel). 2023 Jun 7;13(6):1333. doi: 10.3390/life13061333 (PMC10300762; doi:10.3390/life13061333)

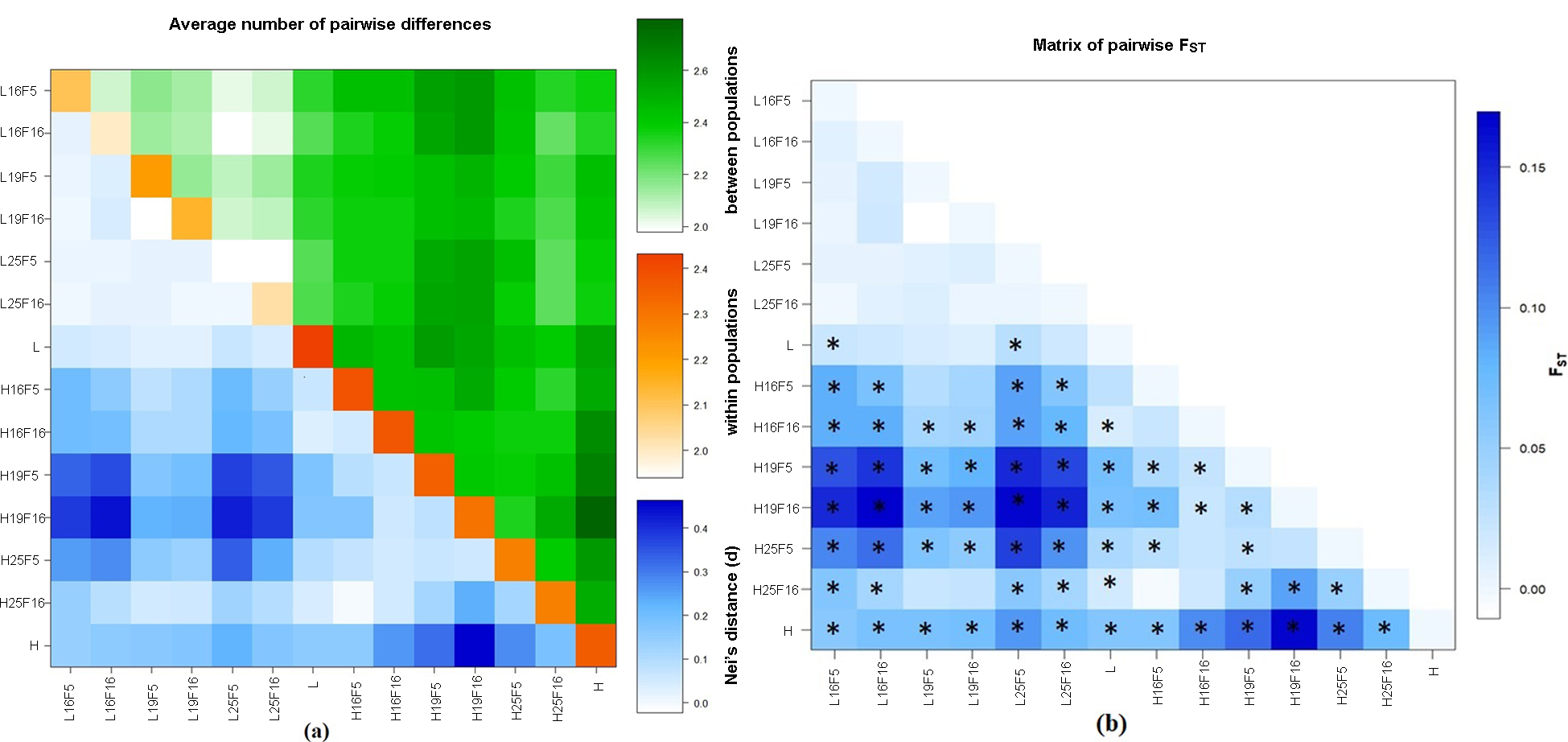

Supplement: Supplementary file 1 [file life-13-01333-s001.zip › Figure 1.tif]

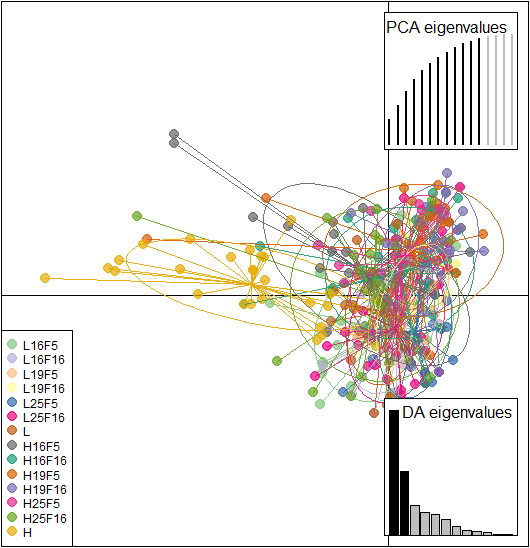

Supplement: Supplementary file 1 [file life-13-01333-s001.zip › Figure 2.png]

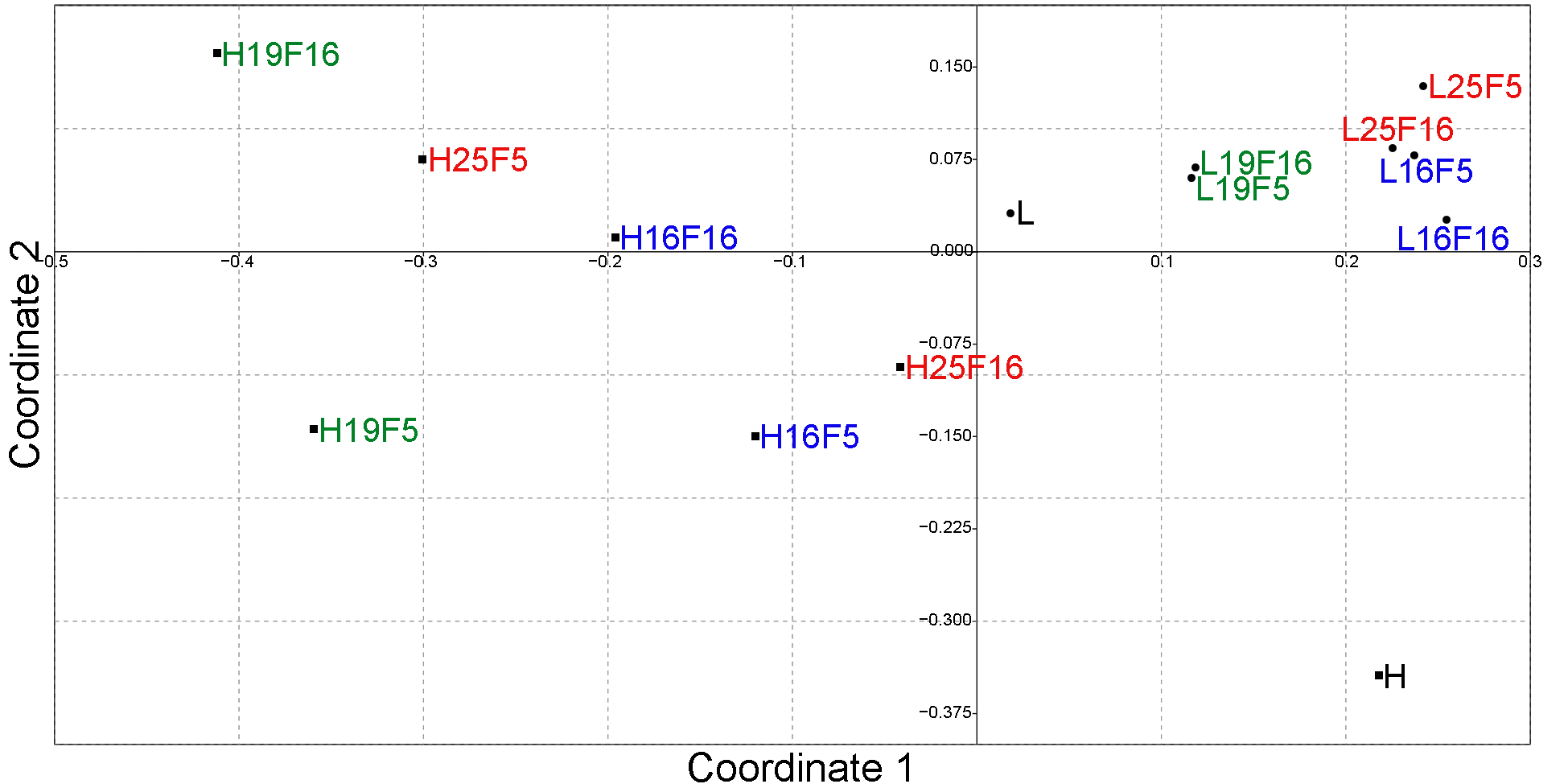

Supplement: Supplementary file 1 [file life-13-01333-s001.zip › Figure 3.tif]

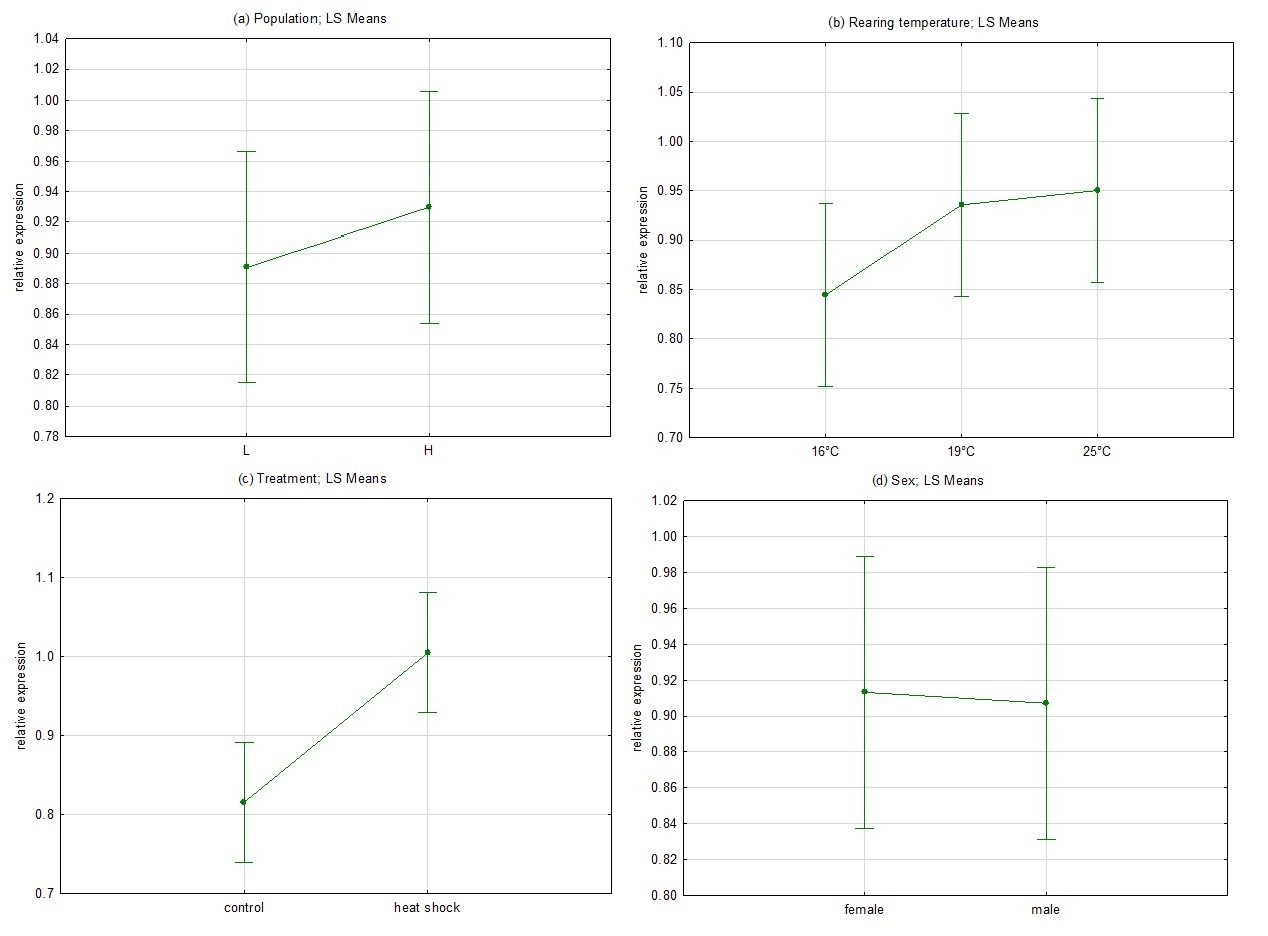

Supplement: Supplementary file 1 [file life-13-01333-s001.zip › Figure 4.jpg]

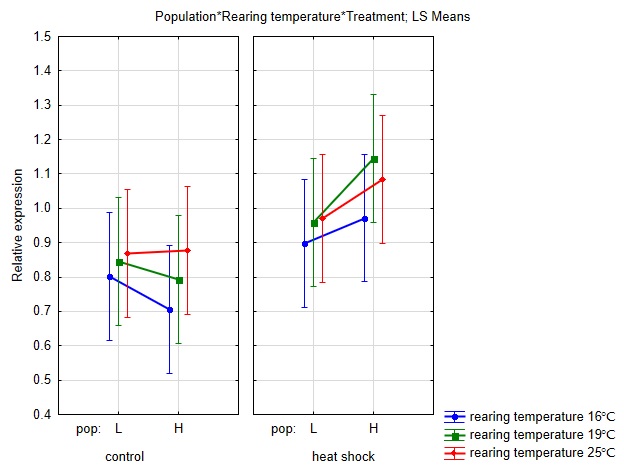

Supplement: Supplementary file 1 [file life-13-01333-s001.zip › Figure 5.jpg]
